# Generation and intracellular trafficking of a polysialic acid-carrying fragment of the neural cell adhesion molecule NCAM to the cell nucleus

**DOI:** 10.1038/s41598-017-09468-8

**Published:** 2017-08-17

**Authors:** Nina Westphal, Gabriele Loers, David Lutz, Thomas Theis, Ralf Kleene, Melitta Schachner

**Affiliations:** 1 Zentrum für Molekulare Neurobiologie, Universitätsklinikum Hamburg-Eppendorf, Falkenried 94, 20251 Hamburg, Germany; 2 Institut für Strukturelle Neurobiologie, Universitätsklinikum Hamburg-Eppendorf, Falkenried 94, 20251 Hamburg, Germany; 3 Center for Neuroscience, Shantou University Medical College, 22 Xin Ling Road, Shantou, Guangdong 515041 China; 4 Keck Center for Collaborative Neuroscience and Department of Cell Biology and Neuroscience, Rutgers University, 604 Allison Road, Piscataway, NJ 08854 USA

## Abstract

Polysialic acid (PSA) and its major protein carrier, the neural cell adhesion molecule NCAM, play important roles in many nervous system functions during development and in adulthood. Here, we show that a PSA-carrying NCAM fragment is generated at the plasma membrane by matrix metalloproteases and transferred to the cell nucleus via endosomes and the cytoplasm. Generation and nuclear import of this fragment in cultured cerebellar neurons is induced by a function-triggering NCAM antibody and a peptide comprising the effector domain (ED) of myristoylated alanine-rich C kinase substrate (MARCKS) which interacts with PSA within the plane of the plasma membrane. These treatments lead to activation of the fibroblast growth factor (FGF) receptor, phospholipase C (PLC), protein kinase C (PKC) and phosphoinositide-3-kinase (PI3K), and subsequently to phosphorylation of MARCKS. Moreover, the NCAM antibody triggers calmodulin-dependent activation of nitric oxide synthase, nitric oxide (NO) production, NO-dependent S-nitrosylation of matrix metalloprotease 9 (MMP9) as well as activation of matrix metalloprotease 2 (MMP2) and MMP9, whereas the ED peptide activates phospholipase D (PLD) and MMP2, but not MMP9. These results indicate that the nuclear PSA-carrying NCAM fragment is generated by distinct and functionally defined signal transducing mechanisms.

## Introduction

In mammals, NCAM is the predominant carrier of PSA, a polymer of α2,8-linked sialic acid monomers. PSA and its carrier NCAM play important roles not only during development, but also in adult central and peripheral nervous system by regulating cell interactions and by affecting synaptic activities and regeneration after trauma^1–7^. PSA modulates the functions of NCAM and influences cell interactions by direct binding to histone H1, brain-derived neurotrophic factor (BDNF), FGF-2 and MARCKS^8–11^.

Proteolytic processing of NCAM by different proteases at the cell surface modulates cell interactions, and the resulting fragments influence several cellular events, such as neurite outgrowth^12–17^. We had found that PSA-lacking and -carrying proteolytic NCAM fragments comprising the intracellular and transmembrane domains as well as part of the extracellular domain enter the cell nucleus after their generation at the plasma membrane^18, 19^. The PSA-lacking transmembrane NCAM fragment is generated by a serine protease at the plasma membrane upon stimulation of cultured cerebellar neurons or NCAM-expressing transfected CHO cells with surrogate ligands, e.g. function-triggering NCAM antibody, and reaches the cell nucleus via the endoplasmic reticulum and cytoplasm in a calmodulin-dependent manner^18^. In the present study, we addressed the question by which mechanisms and pathways the PSA-carrying transmembrane NCAM fragment is generated and reaches the nucleus. Our results show that generation and nuclear import of the PSA-carrying and PSA-lacking NCAM fragments are mediated by different mechanisms.

## Results

### Generation of the nuclear PSA-carrying NCAM fragment involves MMP2 and MMP9

**Figure 1:**
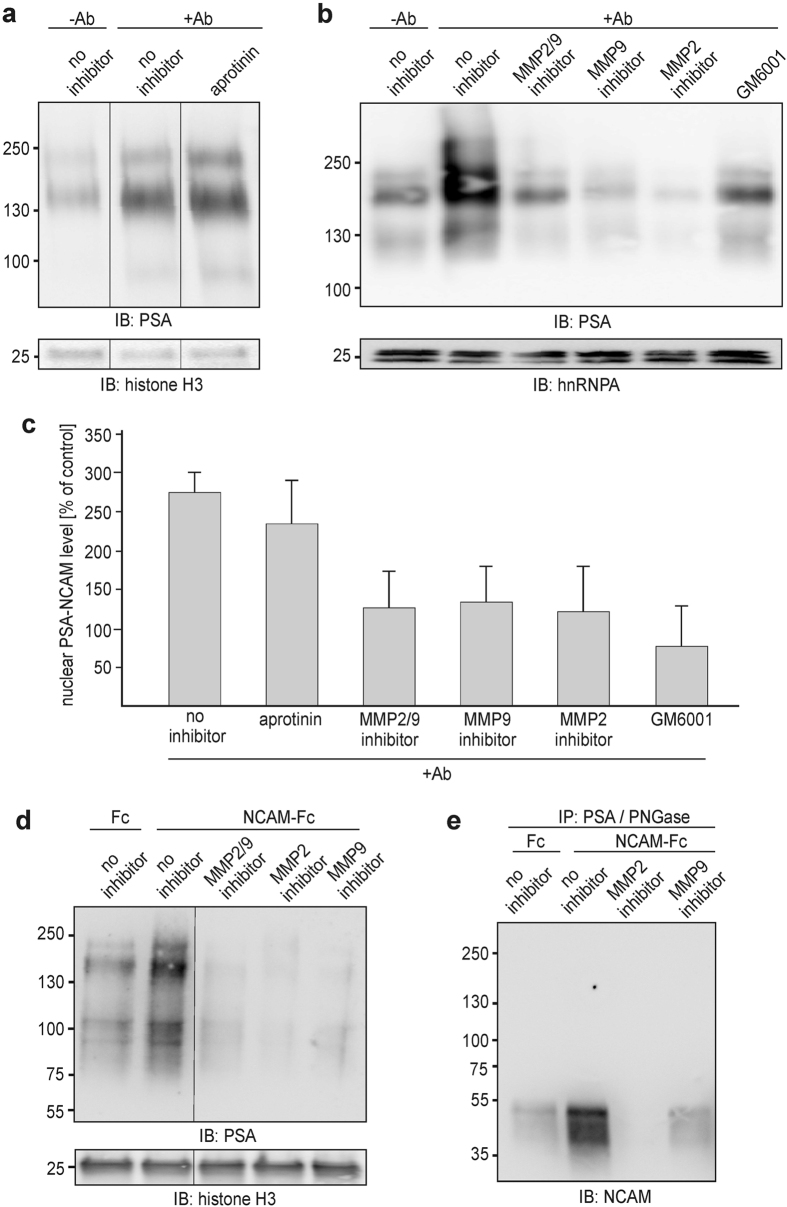
Generation and nuclear import of the PSA-carrying NCAM fragment depends on metalloproteases. (**a**–**e**) Wild-type cerebellar neurons were pre-treated without (no inhibitor) or with aprotinin (1 µM), GM6001 (100 nM), and MMP2 inhibitor (25 nM), MMP9 inhibitor (10 nM) or MMP2/MMP9 inhibitor (MMP2/9) (100 µM) and then incubated without (−Ab) and with chicken NCAM antibody (12.5 µg/ml IgY) (+Ab) (**a**–**c**) or with NCAM-Fc (10 µg/ml) or Fc (2 µg/ml) (**d**,**e**). Nuclear fractions were subjected to immunoblot (IB) analysis with the PSA antibody or with histone H3 or hnRNPA antibody to control for loading (**a**,**b**,**d**) or to immunoprecipitation (IP) with PSA antibody, treatment of the PSA immunoprecipitates with PNGase F and immunoblot analysis of the N-deglycosylated proteins with a rabbit antibody against the extracellular NCAM domain (**e**). (**a**,**b**,**d**,**e**) Representative immunoblots out of three experiments are shown. Lanes that were not adjacent to each other but derived from the same blot are indicated by dividing lines. (**c**) Nuclear PSA levels were quantified by densitometry and normalized to the histone H3 levels. Mean values with standard deviation from 3 independent experiments are shown for the PSA levels relative to the PSA level of non-treated cells (set to 100%).

After having shown that NCAM fragments with or without PSA enter the nucleus^18, 19^, we here tested whether these fragments are generated and transferred to the nucleus by the same or different mechanisms. Since the PSA-lacking NCAM fragment is generated by a serine protease^18^, we first analysed whether the nuclear PSA-carrying NCAM fragment derives from proteolytic cleavage by a serine protease activity. Hence, nuclear fractions were subjected to immunoblot analysis with PSA-specific antibody after treatment of cultured cerebellar neurons with a function triggering NCAM antibody in the absence or presence of the serine protease inhibitor aprotinin, which inhibits the generation of the PSA-lacking transmembrane NCAM fragment^18^. The nuclear PSA-NCAM levels were enhanced after stimulation with NCAM antibody in the absence and presence of aprotinin relative to the nuclear levels of non-stimulated neurons (Fig. 1a,c), indicating that serine proteases are not involved in the generation of the PSA-carrying NCAM fragment. Since *in vitro* and *in vivo* studies have shown cleavage of NCAM by MMP2 and MMP9^16, 17^, we determined whether these proteases are involved in the generation of the nuclear PSA-carrying NCAM fragment using the broad range metalloprotease inhibitor GM6001 or inhibitors specific for MMP2 and/or MMP9. In the presence of either inhibitor, nuclear PSA-NCAM levels were not significantly enhanced by the NCAM antibody treatment relative to the nuclear levels of non-stimulated neurons and in contrast to the enhanced levels observed after NCAM antibody stimulation in the absence of inhibitors (Fig. 1b,c). Similarly, nuclear PSA-NCAM levels were increased by stimulation with NCAM-Fc in the absence of inhibitors relative to Fc treatment, whereas no enhancement in nuclear PSA-NCAM levels was observed after NCAM-Fc treatment in the presence of MMP2-, MMP9- or MMP2/9-specific inhibitors (Fig. 1d). To substantiate that the nuclear PSA is indeed attached to the NCAM fragment that is generated by MMP2- and MMP9-mediated cleavage, we performed immunoprecipitations with PSA antibody using the nuclear fractions from cultured cerebellar neurons after treatment with NCAM-Fc in the absence and presence of MMP2 or MMP9 inhibitors and treated the immunoprecipitates with peptide-N-glycosidase F to remove N-glycans including PSA. Levels of the major N-deglycosylated NCAM fragment were higher in immunoprecipitates from neurons stimulated with NCAM-Fc in the absence of inhibitors than in those treated with Fc, whereas levels of the N-deglycosylated NCAM fragment were not increased upon NCAM antibody stimulation in the presence of the MMP2- or MMP9-specific inhibitors (Fig. 1e).

In summary, these results indicate that MMP2 and MMP9 are both involved in the generation of the PSA-carrying NCAM fragment and that activation of these MMPs and generation of the PSA-carrying NCAM fragment are triggered by binding of a surrogate NCAM ligand, e.g. NCAM antibody, and the homophilically acting NCAM-Fc to PSA-NCAM.

### The PSA-carrying NCAM fragment is generated at the plasma membrane and is transferred to the nucleus via endosomes and the cytoplasm

**Figure 2:**
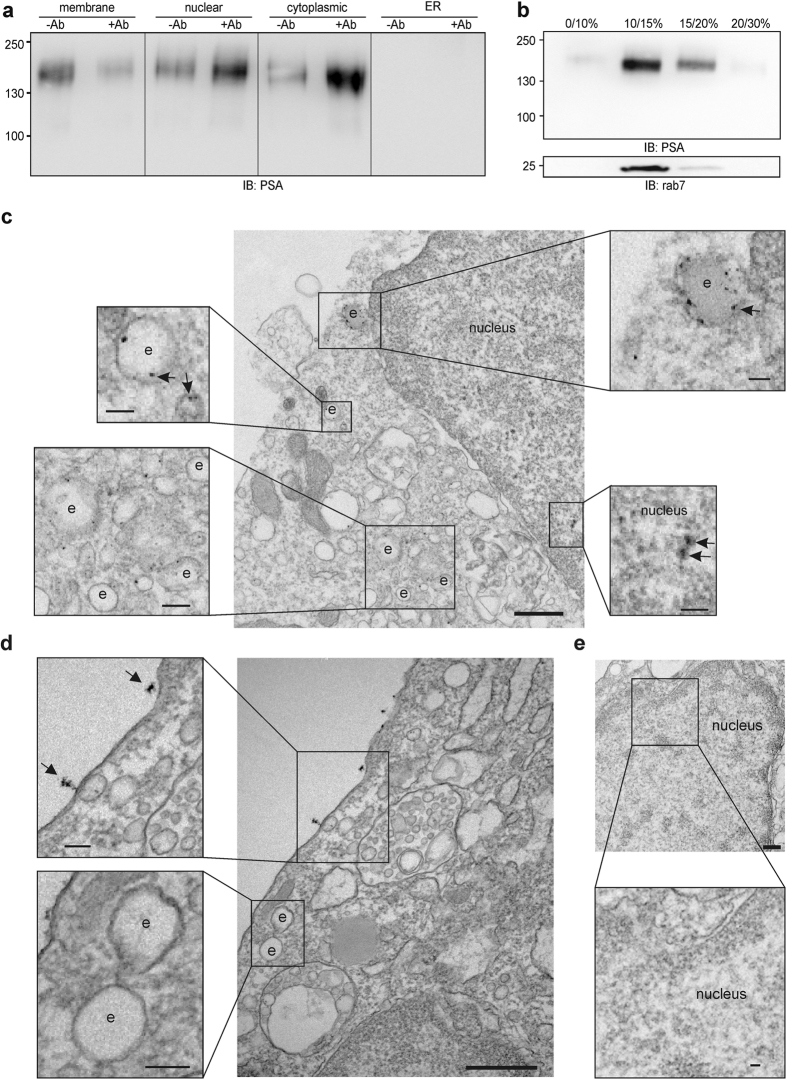
The PSA-carrying NCAM fragment is transported from the cell surface to the nucleus via endosomes. (**a**,**b**) PSA-NCAM-expressing CHO cells were subjected to cell surface biotinylation, stimulation with chicken NCAM antibody (12.5 µg/ml IgY) and subcellular fractionation. Biotinylated proteins from membrane, cytoplasmic, nuclear and ER fractions (**a**) and from fractions of an iodixanol step gradient (**b**) were subjected to immunoblot analysis with the PSA antibody or rab7 antibody. Representative immunoblots out of three independent experiments are shown. Lanes that were not adjacent to each other but derived from the same blot are indicated by dividing lines. (**c**–**e**) Representative electron microscopic images of a cerebellar neuron after treatment with chicken NCAM antibody (**c**) and of a non-treated neuron (**d**,**e**) are shown (Scale bar: 500 nm). Arrows indicate PSA-NCAM detected by immunogold grains in nuclei and endosome-like structures (**c**) or at the cell surface (**d**). (**e**) Part of the nucleus of a non-treated neuron is shown. (**c**–**e**) Boxes indicate sections at higher magnification and scale bars in the boxes represent 100 nm.

Next, we addressed the question by which pathway the PSA-carrying NCAM fragment is transported to the cell nucleus. To monitor the PSA-NCAM fragment on its way from the plasma membrane to the nucleus, PSA-NCAM-expressing CHO cells were subjected to cell surface biotinylation and treatment without or with NCAM antibody followed by subcellular fractionation. Thereafter, biotinylated proteins were isolated from the fractions and subjected to immunoblot analysis with the PSA antibody. The levels of biotinylated PSA-NCAM in plasma membrane-enriched fractions were reduced after NCAM antibody stimulation relative to the levels in fractions from non-treated cells, while the levels of biotinylated cell surface-derived PSA-NCAM in nuclear and cytoplasmic fractions were enhanced after antibody treatment (Fig. 2a), indicating that the PSA-NCAM fragment is generated at the plasma membrane and transported to the nucleus via the cytoplasm. Since the PSA-lacking NCAM fragment is translocated to endosomes and the endoplasmic reticulum (ER) after its generation at the plasma membrane^18^, we also tested fractions enriched in ER or endosomes for the presence of biotinylated PSA-NCAM. No biotinylated PSA-NCAM was detectable in ER-enriched fractions (Fig. 2a), while biotinylated cell surface-derived PSA-NCAM was found in a fraction showing high levels of the late endosomal marker rab7 (Fig. 2b). These results indicate that the PSA-carrying NCAM fragment is transferred to endosomes, but not to the ER after its generation at the plasma membrane.

Immunogold staining and electron microscopy revealed high levels of PSA-NCAM in oval or round late endosome-like structures of ~100 to ~400 nm^20^ and gold grain clusters in nuclei after NCAM antibody stimulation (Fig. 2c). In contrast, PSA-NCAM was hardly detectable in endosome-like structures or nuclei of non-stimulated neurons (Fig. 2d,e). However, clusters of PSA-NCAM were observed at the cell surface of non-stimulated neurons, but not of neurons treated with the NCAM antibody (Fig. 2c,d). These results support the notion that the PSA-NCAM fragment is translocated from the plasma membrane to the nucleus via endosomes.

**Figure 3:**
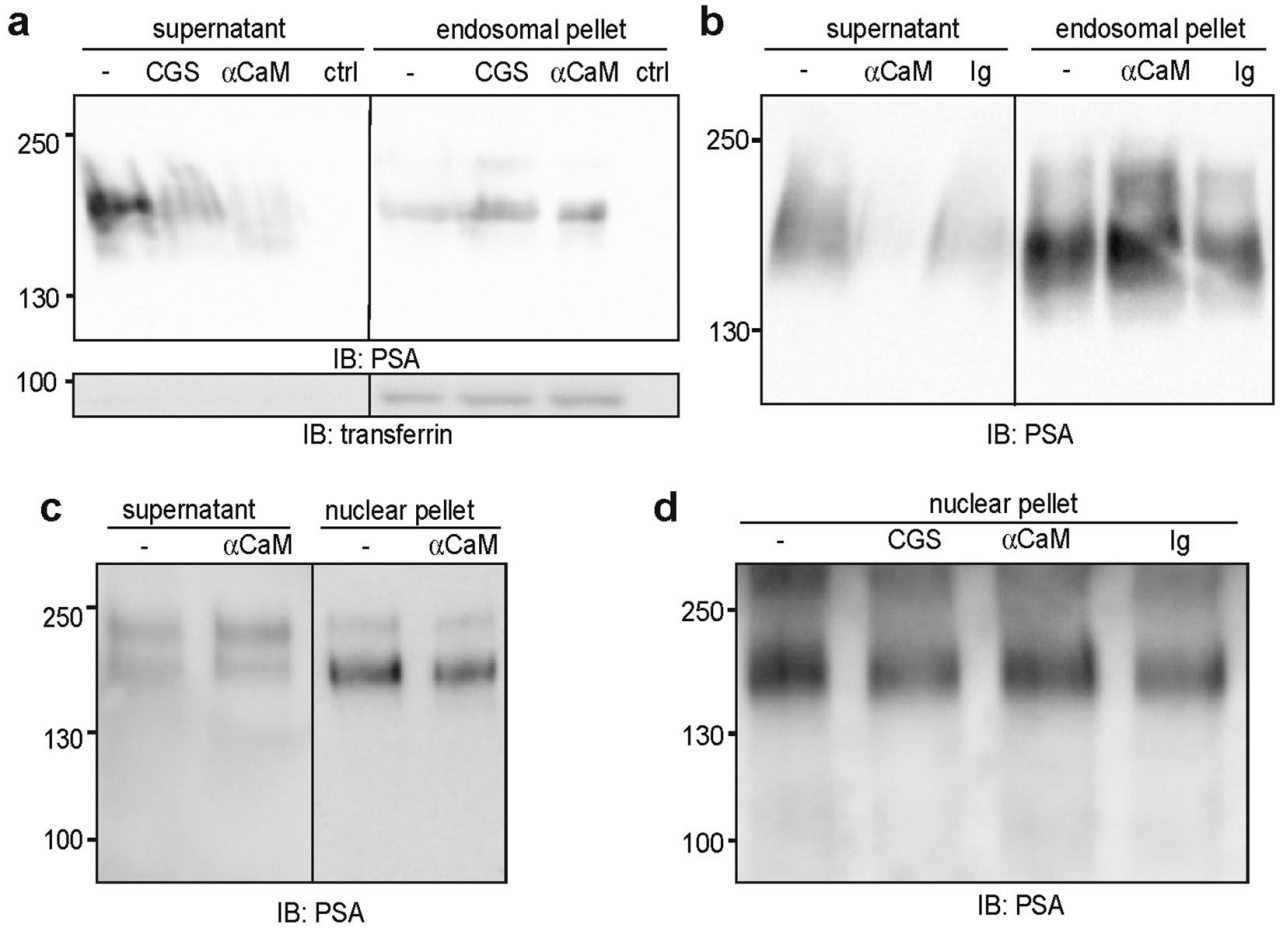
The release of the PSA-carrying NCAM fragment from endosomes is mediated by calmodulin, while the nuclear import is not mediated by calmodulin. (**a**,**b**) A cytoplasmic fraction was isolated from untreated PSA-deficient CHO cells and an endosome-enriched fraction was isolated from PSA-expressing CHO cells after cell surface biotinylation and treatment with NCAM antibody. The cytoplasmic fraction was incubated with buffer (ctrl) or with the endosomal fraction in the absence (−) or presence of 10 µM calmodulin inhibitor CGS9343B (CGS), 8 µg/ml calmodulin antibody (αCaM) or 8 µg/ml of a non-immune control antibody (Ig). After ultracentrifugation, biotinylated proteins were isolated from the resulting pellets and supernatants and subjected to immunoblot analysis with the PSA antibody. Before isolation of biotinylated proteins, aliquots of the pellets and supernatants were subjected to immunoblot analysis with transferrin antibody to check for integrity of endosomes. (**c**,**d**) Nuclei were isolated from NCAM-deficient cerebellar neurons and a cytoplasmic fraction was isolated from wild-type cerebellar neurons after treatment with chicken NCAM antibody (12.5 µg/ml IgY). Nuclei were incubated with buffer (ctrl) or with the cytoplasmic fraction in the absence or presence of 10 µM calmodulin inhibitor CGS9343B (CGS) or 8 µg/ml calmodulin antibody (αCaM) or 8 µg/ml control antibody (Ig) followed by centrifugation at 1,000 × g. The pellets and supernatants were subjected to immunoblot analysis with the PSA antibody. (**a**–**d**) Representative immunoblots out of two independent experiments are shown. Lanes that were not adjacent to each other but derived from the same blot are indicated by dividing lines.

By *in vitro* translocation assays^18, 21^ we analysed whether the PSA-NCAM fragment is released from endosomes into the cytoplasm and imported from the cytoplasm into nuclei. Since calmodulin mediates the release of the PSA-lacking NCAM fragment from the ER membrane into the cytoplasm and is required for the import of this fragment from the cytoplasm into the nucleus^18^, we in parallel used a function-blocking calmodulin antibody and the calmodulin inhibitor CGS9343B^18^ to test whether the release of the PSA-carrying NCAM fragment from endosomes and the nuclear import of this fragment depend on calmodulin. To analyse the release from endosomes into the cytoplasm, a fraction enriched in endosomes was isolated from PSA-NCAM-expressing CHO cells after cell surface biotinylation and NCAM antibody treatment and was incubated with a cytoplasmic fraction from PSA-lacking NCAM-expressing CHO cells in the absence or presence of the calmodulin antibody or inhibitor. Immunoblot analysis with an antibody against the endosomal cargo marker transferrin showed the intactness of the endosomes after incubation and centrifugation (Fig. 3a). The samples were then subjected to ultracentrifugation followed by isolation of biotinylated proteins from the resulting pellets and supernatants as well as immunoblot analysis of the biotinylated proteins with the PSA antibody. In the presence of the calmodulin antibody or inhibitor, the PSA-NCAM levels in the supernatants were lower and the levels in the pellets were higher than those in the absence of the calmodulin antibody and inhibitor (Fig. 3a), indicating that the calmodulin antibody and inhibitor blocked the release of PSA-NCAM from endosomes. In contrast to the calmodulin antibody, a non-immune control antibody only slightly affected the release of PSA-NCAM from endosomes (Fig. 3b). These results suggest that PSA-NCAM is released from the endosomal membrane in a calmodulin-dependent manner.

To investigate the import of PSA-NCAM from the cytoplasm into the nucleus, a cytoplasmic fraction was isolated from cultured wild-type cerebellar neurons after NCAM antibody treatment and incubated with nuclei of PSA-NCAM-lacking cerebellar neurons from NCAM-deficient mice in the absence or presence of the calmodulin antibody. After incubation, the samples were centrifuged and the resulting pellet and supernatant fractions were subjected to immunoblot analysis with the PSA antibody. The PSA-NCAM levels in the supernatant and pellet fractions in the absence of the calmodulin antibody were only slightly higher and lower, respectively, when compared to the levels in the absence of antibody (Fig. 3c), indicating that the calmodulin antibody does not inhibit the nuclear import of PSA-NCAM. Similarly, the calmodulin inhibitor and a non-immune control antibody also did not affect the nuclear import of PSA-NCAM (Fig. 3d). These results indicate that the PSA-NCAM fragment is imported from the cytoplasm into the nucleus and that calmodulin plays no or only a minor role in this process.

### The NCAM antibody-triggered generation of the PSA-carrying NCAM fragment depends on calmodulin and on the activation of the FGF receptor, PLC and PKC

**Figure 4:**
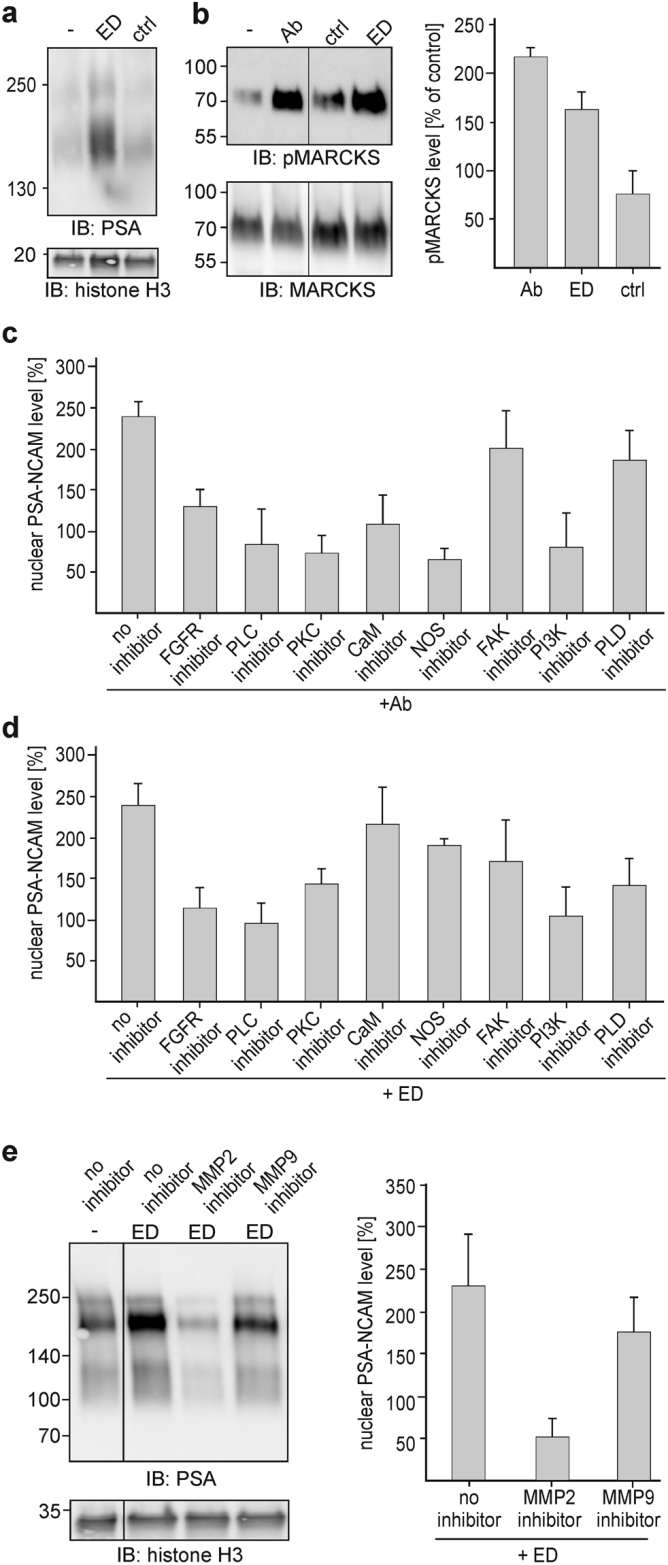
NCAM antibody and the MARCKS-derived ED peptide trigger the generation and nuclear import of PSA-NCAM by different pathways. (**a**–**e**) Cerebellar neurons were treated without (−) (**a**,**b**,**e**) or with chicken NCAM antibody (12.5 µg/ml IgY) (Ab) (**b**,**c**), ED peptide (25 µg/ml) (ED) (**a**,**b**,**d**,**e**) or ED control peptide (25 µg/ml) (ctrl) (**a**,**b**). (**a**) Nuclear fractions were isolated and subjected to immunoblot analysis with PSA antibody or with histone H3 antibody to control loading. (**b**) Cell lysates were probed by immunoblot analysis with an antibody against phosphorylated MARCKS (pMARCKS) or total MARCKS. Levels of phosphorylated MARCKS were quantified by densitometry and normalized to the levels of total MARCKS. Mean values with standard deviation from 3 independent experiments are shown for the levels of phosphorylated MARCKS in treated neurons relative to the levels in non-treated cells (set to 100%). (**c**,**d**) Cerebellar neurons were pretreated without (no inhibitor) or with inhibitors of FGF receptor (100 nM), calmodulin (10 µM), PLC (10 µM), PKC (1.5 µM), NOS (100 µM), FAK (10 µM), PLD (40 nM) or PI3K (4 µM) and then treated without (−) or with chicken NCAM antibody (12.5 µg/ml IgY) (Ab) (**c**) or ED peptide (25 µg/ml) (ED) (**d**) or were pretreated without (no inhibitor) or with inhibitors of MMP2 (25 nM) or MMP9 (10 nM) and then treated with the ED peptide (25 µg/ml) (**e**). Nuclear fractions were subjected to immunoblot analysis with the PSA-specific antibody or histone H3 antibody to control loading. Nuclear PSA levels were quantified by densitometry and normalized to the histone H3 levels. Mean values with standard deviation from 3 independent experiments are shown for the normalized PSA levels in treated cells relative to the PSA level of non-treated cells (set to 100%). (**a**,**b**,**e**) Representative immunoblot out of three experiments is shown. Lanes that were not adjacent to each other but derived from the same blot are indicated by dividing lines.

Since PSA interacts with the effector domain of MARCKS in the plane of the plasma membrane and since a synthetic peptide comprising the effector domain (ED peptide) binds to PSA^9^, we investigated whether the MARCKS-derived PSA-binding peptide triggers the generation of the nuclear PSA-carrying NCAM fragment. Thus, we treated cerebellar neurons with the ED peptide and the ED control peptide, which does not bind to PSA. Relative to the nuclear PSA-NCAM fragment level of non-treated cells, the nuclear PSA-NCAM levels were enhanced by treatment of neurons with the ED peptide, but not by the ED control peptide (Fig. 4a). Quantification of the nuclear PSA-NCAM levels from independent experiments revealed that the ED peptide-triggered increase of nuclear PSA-NCAM levels (219 ± 36%; n = 8; p < 0.005, one-way ANOVA with Dunnett’s multiple comparison test) was very similar to the increase triggered by the NCAM antibody (235 ± 58%; n = 11; p < 0.001) (data not shown). This result shows that the ED peptide triggers the generation of the PSA-carrying NCAM fragment like the NCAM antibody.

Non-phosphorylated MARCKS, which is predominantly found at the plasma membrane, binds to calmodulin and acts as a reservoir of ‘inactive’ calmodulin^22, 23^. Phosphorylation of MARCKS by PKC leads to the release of MARCKS from the plasma membrane into the cytoplasm and to the dissociation of calmodulin from MARCKS^23^. To test whether phosphorylation of MARCKS and the subsequent release of calmodulin from MARCKS are required for the generation of the PSA-NCAM fragment, we determined the level of phosphorylated MARCKS as an indicator of the level of free ‘active’ calmodulin in the cytoplasm. Relative to the level in non-stimulated neurons, the levels of phosphorylated MARCKS were increased after stimulation with the NCAM antibody and the ED peptide, but not with the ED control peptide (Fig. 4b). This result suggests that NCAM antibody and ED peptide trigger the phosphorylation of MARCKS and the dissociation of calmodulin from MARCKS.

Since surrogate NCAM ligands trigger co-signalling with the FGF receptor^24^ which in turn activates PLC and leads to activation of PKC^25^, we used inhibitors of the FGF receptor, PLC and PKC to analyse whether the generation of the PSA-carrying fragment depends on activation of the FGF receptor, PLC or PKC. In the presence of these inhibitors, nuclear PSA-NCAM levels were not enhanced by NCAM antibody or ED peptide treatment when compared to the levels after treatment without inhibitors (Fig. 4c,d). These results indicate that the generation of the PSA-carrying NCAM fragment by NCAM antibody and ED peptide depends on the activation of FGF receptors and subsequent activation of PLC and PKC.

MARCKS sequesters phosphatidylinositol-4,5-bisphosphate (PIP2)^26–29^ which is not only the substrate of PLC, but also serves as a docking platform and substrate for activated PI3K. Since phosphorylation of MARCKS by PKC leads to liberation of sequestered PIP2 and to activation of PI3K^30, 31^, we determined whether NCAM antibody- and ED peptide-triggered generation of the PSA-NCAM fragment depend on the activation of PI3K using an inhibitor of PI3K and found that the inhibitor blocked the generation of the nuclear PSA-carrying NCAM fragment by either stimulation (Fig. 4c,d).

Given that the generation and nuclear import of PSA-lacking NCAM fragment depend on calmodulin and activation of fyn/fak^18^, we investigated whether the enhancement of nuclear PSA-NCAM fragment levels by treatment with NCAM antibody or ED peptide was blocked in the presence of the FAK or calmodulin inhibitor. The FAK inhibitor had only a slight effect on the enhancement of nuclear PSA-NCAM levels by either stimulation (Fig. 4c,d). In the presence of the calmodulin inhibitor, the nuclear PSA-NCAM fragment levels were not enhanced after NCAM antibody stimulation when compared to the enhanced levels observed in the absence of inhibitors (Fig. 4c). However, enhanced nuclear PSA-NCAM levels were found in neurons after treatment with the ED peptide in the presence of the calmodulin inhibitor (Fig. 4d). These results indicate that the activation of the fyn/fak pathway is not required for the generation of the PSA-NCAM fragment and that calmodulin is required for the NCAM antibody-triggered, but not for the ED peptide-triggered generation of the PSA-NCAM fragment.

### NCAM antibody-induced generation of the PSA-NCAM fragment depends on NOS-mediated signalling pathways, while ED peptide-induced generation depends on PLD-mediated signalling pathways

Phosphorylation of MARCKS by PKC not only triggers the release of calmodulin from MARCKS but also contributes to activation of calmodulin-mediated signalling, such as activation of NOS^32, 33^. Thus, we investigated whether the calmodulin- and PKC-dependent enhancement of nuclear PSA-NCAM fragment levels by NCAM antibody depends on calmodulin-triggered activation of NOS. In the presence of the NOS inhibitor, nuclear PSA-NCAM levels were not enhanced by NCAM antibody stimulation (Fig. 4c). In contrast, enhanced nuclear PSA-NCAM levels were found in neurons after treatment with the ED peptide in the presence of the NOS inhibitor (Fig. 4d).

**Figure 5:**
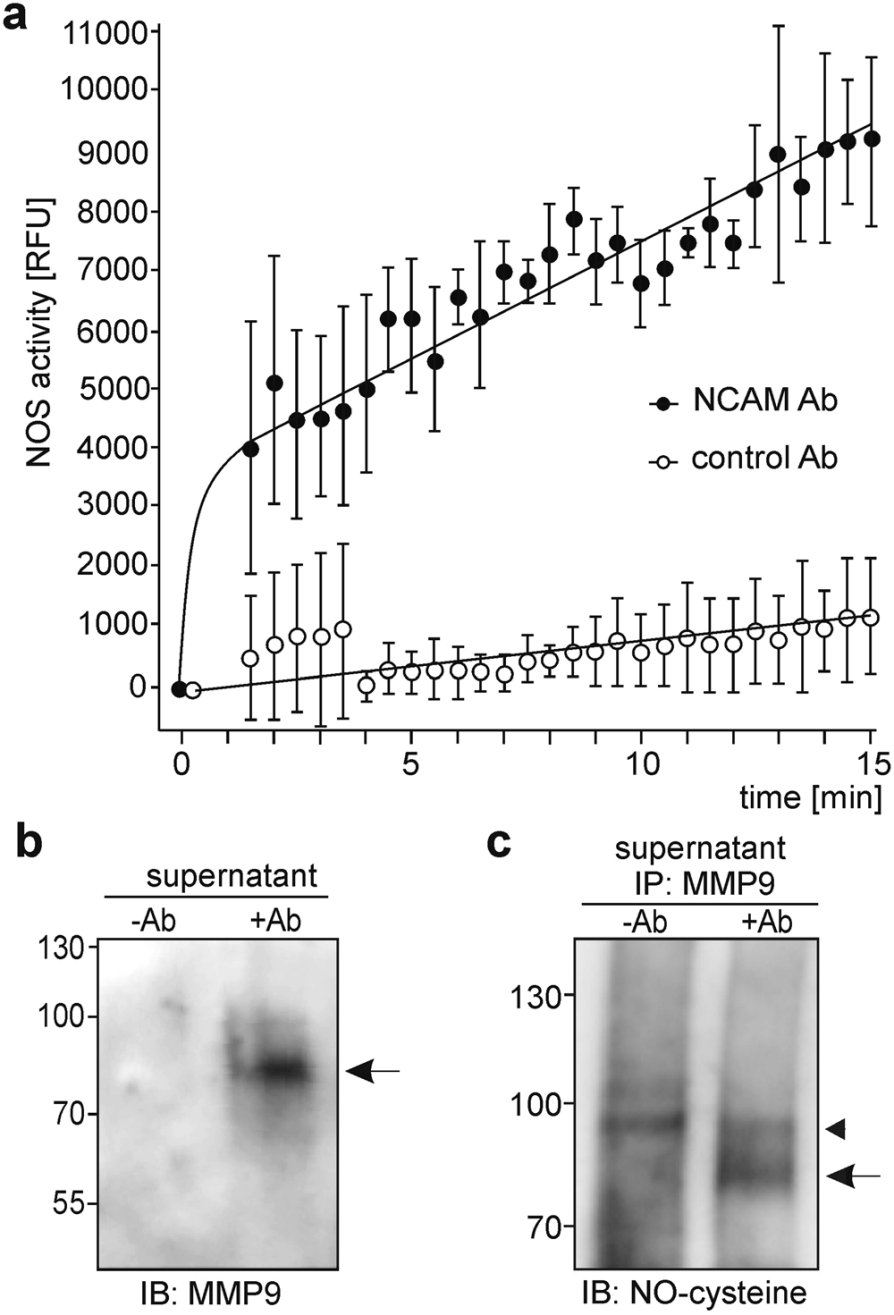
NCAM antibody treatment triggers NO production and S-nitrosylation of MMP9. (**a**) Cerebellar neurons were incubated with guinea pig NCAM antibody (25 µg/ml IgG) or control antibody (25 µg/ml IgG) and the intracellular NO levels were determined before and after antibody treatment (every 30 s for 15 min). Mean values with standard deviation from 3 independent experiments are shown for the NO levels. (**b**,**c**) Cerebellar neurons were incubated without (−Ab) or with guinea pig NCAM antibody (25 µg/ml IgG) (+Ab) for 10 min. Cell culture supernatants were collected and subjected to modification of S-nitrosyl residues of proteins and exchange by biotin (**b**) or to immunoprecipitation with MMP9 antibody followed by immunoblot analysis of the immunoprecipitates with S-nitroso-cysteine antibody (**c**). (**b**) Biotinylated proteins were isolated and probed by immunoblot analysis with MMP9 antibodies for detection of S-nitrosylated MMP9.

To examine whether NCAM antibody treatment of neurons triggers NO synthesis, we determined the intracellular NO levels. A strong increase in NO levels was observed in neurons after addition of the NCAM antibody, whereas only a slight increase was observed when a control antibody was applied (Fig. 5a). These results indicate that the NCAM antibody triggers the activation of NOS and generation of NO, suggesting that NO production is required for the generation of the PSA-NCAM fragment by NCAM antibody treatment.

Since MMP9 is activated by NO via S-nitrosylation of cysteine thiols^34–36^, we tested whether enhanced NO levels after NCAM antibody treatment would lead to enhanced S-nitrosylation of MMP9, which indicates the activation of this protease. To determine extracellular S-nitrosylation of MMP9, culture supernatants from non-treated and NCAM antibody-treated cerebellar neurons were subjected to conversion of S-nitrosylated cysteines into biotinylated cysteines followed by isolation and immunoblot analysis of biotinylated proteins using a MMP9-specific antibody. A biotinylated MMP9-immunopositive band of approximately 80 kDa was detectable in the supernatant from NCAM antibody-treated neurons, but not in supernatant from untreated neurons (Fig. 5b). Culture supernatants from non-treated and NCAM antibody-treated cerebellar neurons were also subjected to immunoprecipitation using a MMP9 antibody and to immunoblot analysis with antibody against S-nitroso-cysteine. An S-nitroso-cysteine-immunopositive band of approximately 80 kDa was only detectable in the MMP9 immunoprecipitate from supernatants of NCAM antibody-treated neurons (Fig. 5c). These results suggest that NCAM antibody enhances MMP9 activity via S-nitrosylation of MMP9.

Since the ED peptide triggers the generation of the PSA-carrying NCAM fragment via calmodulin- and NOS-independent pathways, we examined the ED peptide-induced generation of the PSA-NCAM fragment in more detail. First, we examined whether the ED peptide triggers the cleavage of PSA-NCAM by MMP9 and MMP2. After treatment with the ED peptide, nuclear PSA-NCAM fragment levels were not enhanced in the presence of the MMP2 inhibitor, while the levels were enhanced in the presence of the MMP9 inhibitor (Fig. 4e), indicating that the ED peptide triggers the activation of MMP2, but not MMP9. Since activation of PLD and generation of phosphatidic acid by PLD regulate production and activity of MMP2^37, 38^, we asked whether the ED peptide triggers PLD-dependent signal pathways to stimulate MMP2-mediated generation of the nuclear PSA-NCAM fragment. To this aim, we treated neurons with ED peptide in the presence or absence of PLD inhibitor and found that this inhibitor blocked the generation of the nuclear PSA-NCAM fragment (Fig. 4d). Interestingly, a less pronounced effect of PLD inhibitor on PSA-NCAM fragment levels were observed after stimulation with NCAM antibody (Fig. 4c), indicating that the NCAM antibody treatment does not trigger the activation of PLD.

## Discussion

**Figure 6:**
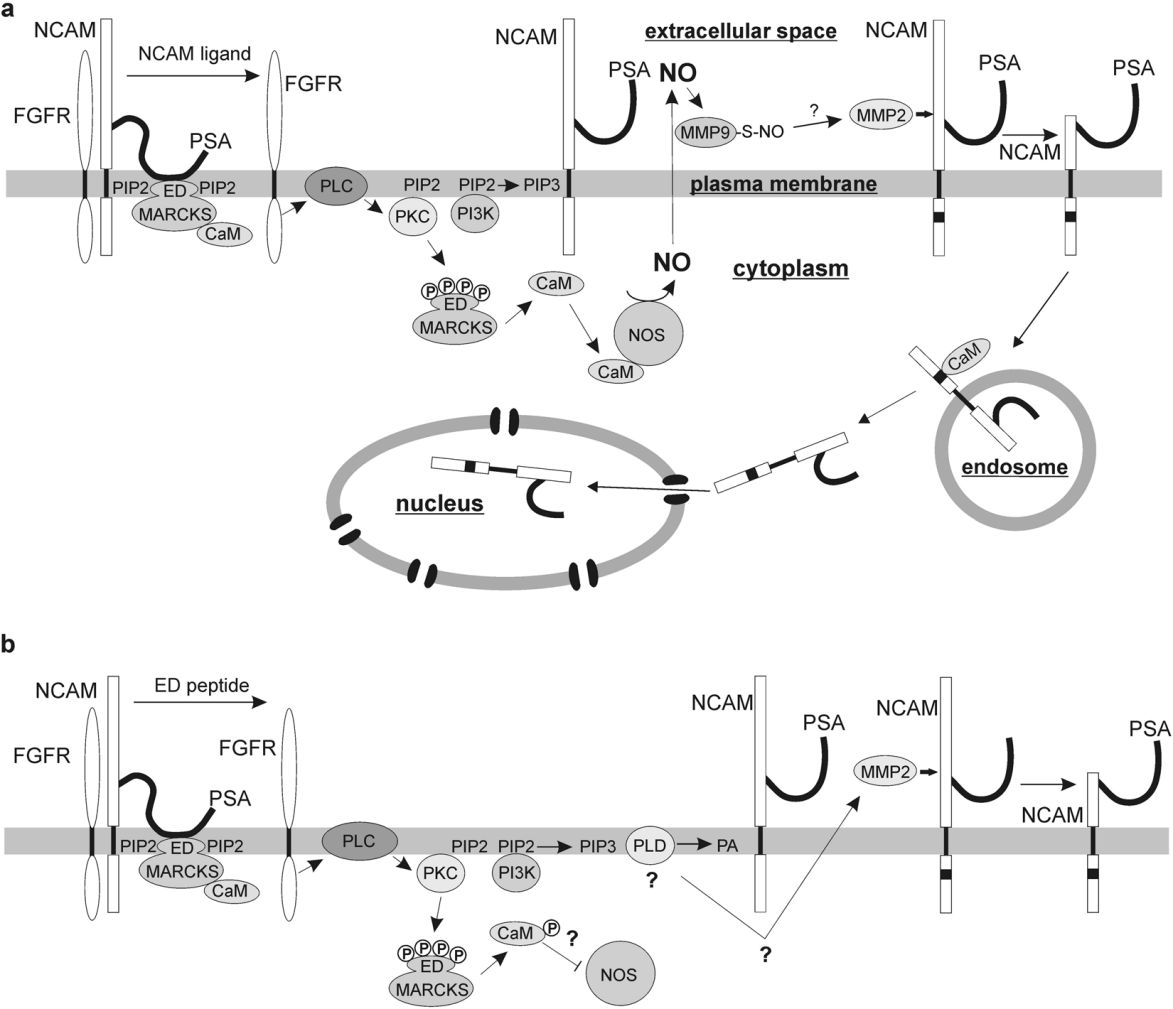
Proposed working model for the generation of the PSA-carrying NCAM fragment. (**a**) PSA-carrying NCAM interacts with the FGF receptor and the effector domain (ED) of non-phosphorylated MARCKS which sequesters calmodulin to the plasma membrane. Binding of NCAM ligands to PSA-NCAM leads to dissociation of the FGF receptor and PSA-NCAM and detachment of the PSA moiety from MARCKS followed by activation of the FGF receptor, PLC, PKC, and PI3K. PKC-mediated phosphorylation of the MARCKS effector domain triggers the release of membrane-bound MARCKS and dissociation of calmodulin from MARCKS. Free calmodulin binds to NOS leading to activation of NOS and production of NO, which is released into the extracellular space. S-nitrosylation of MMP-9 by NO leads to activation of MMP-9 and subsequent activation of MMP2 and cleavage of PSA-NCAM by active MMP2. After cleavage, the PSA-carrying NCAM fragment is internalized, transported to endosomes and released from the endosomal membrane into the cytoplasm involving calmodulin and imported calmodulin-independently from the cytoplasm into the nucleus. (**b**) The MARCKS-derived ED peptide like endogenous stimuli affects the interaction of the PSA moiety and MARCKS and thereby not only triggers the dissociation of the FGF receptor and PSA-NCAM, the activation of the FGF receptor, PLC and PKC and phosphorylation of the MARCKS effector domain, but also the activation of PLD, which produces phosphatidic acid. Activation of PLD and production of phosphatidic acid may lead to inhibition of calmodulin to activate NOS, but triggers the direct activation of MMP2 which cleaves PSA-NCAM.

In previous studies, we showed that PSA-lacking and -carrying NCAM fragments enter the cell nucleus after their generation at the plasma membrane^18, 19^. The PSA-lacking NCAM fragment is generated by a serine protease and the translocation of this fragment from the plasma membrane via the ER and the cytoplasm to the nucleus depends on calmodulin and the fyn/fak pathway^18^. Here, we show that the PSA-carrying NCAM fragment is generated and transported to the nucleus via a pathway which differs from the pathway by which the PSA-lacking NCAM fragment is generated and transported to the nucleus. Based on our results we propose the following sequence of events triggered by NCAM and a surrogate ligand or by the MARCKS-derived ED peptide leading to the generation and nuclear import of the PSA-NCAM fragment. Binding of NCAM or PSA ligands to the NCAM protein backbone or the PSA glycan cause conformational changes and/or interfere with the interaction of NCAM with the FGF receptor leading to activation of the FGF receptor (Fig. 6a,b). Changes in NCAM’s structure and activation of the FGF receptor signalling pathway lead to activation of PLC, PKC and PI3K. PKC-mediated MARCKS phosphorylation results in the dissociation of PSA from MARCKS, detachment of MARCKS from the membrane, and liberation of calmodulin and PIP2 from MARCKS (Fig. 6a,b). Activated PI3K binds to liberated PIP2 and produces the signal molecule PIP3 (Fig. 6a,b). Free calmodulin activates NOS and NO produced by NOS is released into the extracellular space and stimulates the activation of MMP9 and MMP2 (Fig. 6a). Due to the fact that MMP9 is activated by NO via S-nitrosylation of cysteine thiols^34–36^ and our observation that MMP9 is S-nitrosylated upon stimulation with NCAM antibody, it is likely that the attachment of NO to cysteine residues of MMP9 leads to activation of this protease. Based on our results, we propose that NO-dependent activation of MMP9 results in activation of MMP2 and that MMP2 cleaves cell surface full-length PSA-NCAM (Fig. 6a).

The MARCKS-derived ED peptide triggers MMP2-mediated and MMP9-independent cleavage of full-length PSA-NCAM via activation of PLD which produces the signal molecule phosphatidic acid. Activation of PLD depends on PIP3 which is produced from PIP2 by activated PI3K. Since the ED peptide binds to PSA within the plane of the plasma membrane^9^, we suppose that this peptide interferes with the interaction of PSA and MARCKS within the plane of the plasma membrane and thereby triggers PLD-dependent signal transduction pathways leading to the generation of the PSA-NCAM fragment (Fig. 6b), suggesting that the generation of the PSA-NCAM fragment can also be triggered by physiological stimuli which may affect the association of PSA with MARCKS in the plasma membrane. Since the ED peptide-induced generation of the PSA-NCAM fragment is calmodulin- and NOS-independent, it is likely that ED peptide-induced signalling leads to inhibition of the calmodulin-induced activation of NOS and the subsequent NO-dependent activation of MMP9, probably by phosphorylation of calmodulin which blocks NOS activation^39^ (Fig. 6b).

After its generation, the PSA-carrying NCAM fragment is translocated to the nucleus via the endosomal compartment and the cytoplasm. The release of the fragment from the endosomes into the cytoplasm is mediated by calmodulin, while the nuclear import of the fragment is not mediated by calmodulin.

In summary, our results indicate that the generation of the PSA-carrying and PSA-lacking NCAM fragments is mediated by different pathways and mechanisms *in vitro*.

Surrogate PSA-NCAM ligands, e.g. NCAM-Fc, NCAM antibody and the MARCKS-derived ED peptide, used in the present study may mimic the functions of endogenous NCAM ligands and PSA ligands that trigger different signal pathways leading to the generation and nuclear import of the PSA-carrying NCAM fragment *in vivo*. Since soluble extracellular NCAM forms with and without PSA have been documented in the brain and cerebrospinal fluid^40^, it is conceivable that soluble NCAM forms act as PSA-NCAM ligands *in vivo* besides other endogenous ligands. Soluble NCAM forms with and without PSA may bind to the protein core of PSA-NCAM and thereby trigger NO-dependent, MMP9-mediated generation of the PSA-NCAM fragment, while the PSA moiety of soluble PSA-NCAM forms may interfere with the interaction of membrane-bound PSA-NCAM and MARCKS within the plasma membrane and thereby triggers NO-independent, MMP2-mediated generation of the PSA-NCAM fragment. Since NO regulates numerous processes in the nervous system including synaptic plasticity and control of circadian rhythm^41, 42^, NCAM-induced NO production may be involved in regulation of NO-dependent processes. MMP2 and MMP9 which have numerous substrates play important roles in the nervous system during development and in the adulthood as well as after injury and have been implicated in neurological disorders and diseases^43^. Thus, the activation of MMP9 and MMP2 through NCAM-induced signal pathways not only leads to cleavage of NCAM but also to cleavage of other cell surface and extracellular matrix proteins and may contribute to PSA-NCAM’s role in regulation of processes in normal, injured or diseased developing and adult nervous system. In this context it is important to mention that elevated levels of soluble NCAM have been found in brains or cerebrospinal fluids of patients with Alzheimer disease, schizophrenia or medulloblastoma^40^, and that overexpression of soluble extracellular NCAM in mice causes defects in synaptic connectivity and produces abnormal behaviour relevant in schizophrenia and other neuropsychiatric disorders^44^. Based on these findings it can be speculated that the PSA-NCAM fragment upon import into the nucleus contributes to PSA-NCAM’s functions in the developing and adult nervous system. Since PSA participates in regulation of cell interactions during development, affects synaptic activities and regeneration after trauma in the adult nervous system and is important in regulating the diurnal internal clock^19^, it is likely that the generation and nuclear import of the PSA-NCAM fragment is required for these PSA-NCAM-mediated functions. Moreover, dysregulated generation and altered nuclear levels of the PSA-NCAM fragment may be involved in the development of neural disorders.

## Methods

### Animals and cell lines

Mice were maintained as described^19^. C57BL/6 J mice or NCAM-deficient mice^45^ and their wild-type littermates of both sexes were used for all experiments which were conducted in accordance with the German and European Community laws on protection of experimental animals and approved by the responsible committee of the State of Hamburg (animal permit number ORG 679 Morph).

PSA-NCAM-expressing CHO cells and PSA-negative NCAM-expressing CHO have been described^46, 47^ and were a gift of Martina Mühlenhoff (Institut für Zelluläre Chemie, Medizinische Hochschule, Hannover, Germany).

### Antibodies and reagents

Production of NCAM-Fc has been described^15, 18^. Polyclonal chicken and guinea pig antibodies against NCAM-Fc were produced by Pineda (Berlin, Germany). Polyclonal rabbit NCAM antibody 1β2 against the extracellular domain has been described^23^. Antibodies against calmodulin (FL-149; sc-5537), transferrin (F-8; sc-373785), hnRNPA (H-200; sc-15385) and histone H3 (C-16; sc-8654) were from Santa Cruz Biotechnology (Heidelberg, Germany). Rabbit polyclonal antibody against MARCKS^48^ was a kind gift of Perry J. Blackshear (Departments of Medicine and Biochemistry, Duke University, Durham, NC, USA). Phospho-MARCKS antibody (Ser152/156; #2741) and Rab7 antibody (D95F2) were from Cell Signaling Technology (New England BioLabs, Frankfurt). The mouse monoclonal PSA antibody 735^49^ was a gift of Rita Gerardy-Schahn (Zentrum Biochemie, Institut für Zelluläre Chemie, Medizinische Hochschule, Hannover, Germany). Rabbit anti-S-nitrosylated-cysteine:UC (SNO-Cys) (ADI-NISC11-A) was from Linaris (Dossenheim, Germany). All secondary antibodies, sera and human Fc were from Dianova (Hamburg, Germany).

Peptide-N-glycosidase F was from New England BioLabs. Protein A/G agarose beads, biotin-5-[(3aS,4 S,6aR)-2-oxo-1,3,3a,4,6,6a-hexahydrothieno[3,4-d]imidazol-4-yl]-N-[6-[3-(pyridin-2-yldisulfanyl) propanoylamino] hexyl]pentanamide (biotin-HDPP), the PLD inhibitor FIPI (CAS939055-18-2) and the PI3K inhibitor LY294002 (CAS154447-36-6) were from Santa Cruz Biotechnology. Neocuproine, methanethiosulfonate (MMTS), sodium ascorbate, the NOS inhibitor N-nitro-L-arginine methyl ester hydrochloride (L-NAME) and the FAK inhibitor 14 (CAS 4506-66-5) were from Sigma-Aldrich. The FGF receptor tyrosine kinase inhibitor PD173074 (CAS 192705-79-6), the PLC inhibitor U-73122 (CAS 112648-68-7) and PKC inhibitor PKC 19-36 (CAS 113731-96-7) were from Tocris (Bristol, UK). The MMP2 Inhibitor III (CAS 704888-90-4), MMP2/MMP9 Inhibitor I (CAS 193807-58-8), MMP9 Inhibitor I (CAS 1177749-58-4), GM6001 (CAS 142880-36-2) and aprotinin were from Merck Chemicals (Darmstadt, Germany). The calmodulin inhibitor CGS9343B was a kind gift of Jacob Zijlstra (Novartis Consumer Health, Rotkreuz, Switzerland). The MARCKS-derived ED peptide (KKKKKRFSFKKSFKLSGFSFKKNKK) and the corresponding control peptide (KKKKKRASAKKSAKLSGASAKKNKK) were purchased from Schafer-N (Copenhagen, Denmark). The NO-indicator 4-amino-5-methylamino-2′,7′-difluorofluorescein diacetate (DAF-AM) was from ThermoFisher Scientific (Darmstadt, Germany).

### Preparation, treatment and subfractionation of cerebellar granule cell cultures

Cerebellar granule neurons were prepared from 6- to 8-day-old C57BL/6 J mice and maintained in poly-L-lysine-coated 6-well plates in 2 ml serum-free medium for 30 h^19^. For pre-treatments, cells were incubated for 1 h with aprotinin (1 µM), GM6001 (100 nM), MMP2 inhibitor III (25 nM), MMP9 inhibitor I (10 nM), MMP2/MMP9 inhibitor I (100 µM), calmodulin inhibitor CGS9343B (10 µM), NOS inhibitor L-NAME (100 µM), FAK inhibitor 14 (10 µM), PKC inhibitor PKC 19–36 (1.5 µM), PLC inhibitor U-73122 (10 µM), PLD inhibitor FIPI (40 nM), PI3K inhibitor LY294002 (4 µM) or FGF receptor inhibitor PD173074 (100 nM) or with phosphate-buffered saline, pH 7.4 (PBS). Cells were then incubated for 30 min without or with chicken or guinea pig NCAM antibody (1:1,000; corresponding to 25 µg/ml IgG or 12.5 µg/ml IgY) or with NCAM-Fc (10 µg/ml), Fc (2 µg/ml), MARCKS-derived ED peptide (25 µg/ml) or control peptide (25 µg/ml) in PBS. Previous experiments showed that the used NCAM antibody and MARCKS-derived ED peptide concentration were optimal for robust and maximal stimulation. For subcellular fractionation the Subcellular Protein Fractionation Kit for Cultured Cells was used (ThermoFisher Scientific).

### Immunoprecipitation and peptide-N-glycosidase F treatment

Immunoprecipitation and peptide-N-glycosidase F treatment of immunoprecipitates were performed as described^18, 19^.

### Immunoblot analysis, immunogold labelling and electron microscopy

Immunoblot analysis and quantification as well as pre-embedding, immunogold labelling and electron microscopic analysis were performed as described^19^. Intensities relative to loading controls were calculated.

### Measurement of NO levels

NO levels of were determined using the NO-indicator DAF-AM^50–52^. Neurons were seeded onto poly-L-lysine-coated black 96-well plates with optical bottom (Greiner Bio-One; Kremsmünster, Austria) and maintained for 29 h. Cells were loaded with DAF-AM (10 µM) for 50 min at 37 °C and 5% CO_2_, washed two times with pre-warmed Krebs-Henseleit buffer (KHB; Sigma-Aldrich) containing additional 2.5 mM CaCl_2_ and 7.5% sodium bicarbonate and incubated with fresh KHB with additives at 37 °C for 10 min. Background NO levels were determined in a multimode plate reader (Tecan Spark 10 M; Tecan, Männedorf, Switzerland) using an excitation of 485 nm and an emission of 520 nm. Cells were then treated with KHB, guinea pig NCAM-antibody (10 µg/well) or control antibody (guinea pig serum, 10 µg/well) and the fluorescence of NO-bound DAF was determined every 30 s for 15 min using the same settings as for the background reading. Fluorescence signals obtained from untreated cells were subtracted from the values obtained for treated cells to control for unspecific NO production. All treatments were performed in triplicates.

### Determination of S-nitrosylation of proteins

A three step biotin switch method^53^ was used for the detection of S-nitrosylated proteins. Cerebellar neurons were maintained in culture medium for 30 h. The culture medium was exchanged against Hanks’ Balanced Salt solution (HBSS) without phenol red and the neurons were treated NCAM or control antibody for 10 min. The cell culture supernatants were collected and 5 ml HEN buffer (25 mM HEPES, pH 7.7, 0.1 mM EDTA, 10 µM neocuproine) containing 2% SDS and 100 µl of 2 M MMTS in dimethylformamide were added to 1 ml of sample and incubated for 20 min at 50 °C to block free thiol groups. The samples were then incubated with 10 ml ice-cold acetone for 30 min at −20 °C and centrifuged at 13,000 × g for 10 min at 4 °C. Pellets were resuspended in 5 ml HENS buffer (HEN buffer containing 1% SDS) and incubated for 1 h at 25 °C with 1 mM sodium ascorbate and 5 mM biotin-HPDP (freshly prepared from a 50 mM stock in dimethylformamide by diluting in dimethyl sulfoxide). After acetone precipitation and centrifugation, the pellets were resuspended in 0.5 ml HENS buffer and 1 ml neutralization buffer (20 mM HEPES pH 7.7, 100 mM NaCl, 1 mM EDTA, 0.5% Triton X-100) and 80 µl streptavidin-coupled magnetic beads (ThermoFisher Scientific) were added. After overnight incubation at 4 °C with rotation, beads were washed 5 times with washing buffer (neutralization buffer containing 600 mM NaCl). The biotinylated proteins were eluted by boiling the beads in 30 µl LDS non-reducing sample buffer (ThermoFisher Scientific) for 10 min at 98 °C.

### Cell surface biotinylation and subcellular fractionation of CHO cells

Cell surface biotinylation of CHO cells was performed as described^18^. Briefly, cells were serum deprived at a confluence of 80% for 16 h, washed thrice with HBSS supplemented with 0.5 mM CaCl_2_ and 2 mM MgCl_2_ (HBSS-2+), and incubated for 1 h at 4 °C with 0.5 mg/ml of sulfo-NHS-LC-biotin (Pierce) in HBSS-2+ . After washing twice with 100 mM glycine, cells were washed with HBSS-2+ and incubated without or with chicken NCAM antibody for 30 min.

The Subcellular Protein Fractionation Kit for Cultured Cells (ThermoFisher Scientific) was used for the isolation of membrane, cytoplasmic and nuclear fractions, and the ER isolation kit (Sigma-Aldrich) was used for the isolation of ER-enriched fractions. The isolation of endosomal fractions was performed as described^18^. Briefly, cells were homogenized and centrifuged at 1,000 × g for 10 min followed by centrifugation of the resulting supernatant at 17,000 × g for 15 min and centrifugation of the 17,000 × g supernatant at 100,000 × g for 1 h. The 100,000 × g pellet was layered on a step gradient of 10, 15, 20 and 30% iodixanol (Axis-Shield, Oslo, Norway) and centrifuged at 100,000 × g for 3 h and the material from the interphases was collected. Isolation of biotinylated proteins using streptavidin-conjugated magnetic beads (Invitrogen) has been described^18^.

### *In vitro* retro-translocation and nuclear import assays

For the analysis of translocation from the endosomal membrane to the cytoplasm, an endosomal fraction isolated from PSA-expressing CHO cells after cell surface biotinylation and treatment with NCAM antibody was incubated with the 100,000 × g cytoplasmic supernatant from untreated PSA-lacking CHO cells for 60 min at 4 °C in translocation buffer (10 mM HEPES, pH 7.2, 40 mM magnesium acetate, 2 mM CaCl_2_, 1 mM DTT, 0.1 mM PMSF) without or with 10 µM CGS9343B or 8 µg/ml calmodulin or control antibody. The samples were then centrifuged at 100,000 × g for 1 h at 4 °C. Biotinylated proteins were isolated from the pellets and supernatants using streptavidin-conjugated magnetic beads and subjected to immunoblot analysis.

For the analysis of nuclear import, untreated NCAM-deficient cerebellar neurons and NCAM antibody-treated wild-type neurons were resuspended in cell lysis buffer (10 mM HEPES, pH 7.5, 10 mM KCl, 0.1% Triton X-100, 1 mM EDTA, 0.1 mM PMSF), homogenized using a Dounce homogenizer, passed through a 27-gauge needle and centrifuged. The 1,000 × g nuclear pellets of untreated NCAM-deficient neurons were incubated with the 100,000 × g cytoplasmic supernatant of NCAM antibody-treated wild-type neurons for 1 h at 37 °C in nuclear translocation buffer (25 mM HEPES, pH 7.4, 12.5 mM KCl, 2.5 mM MgCl_2_, 1.25 mM CaCl_2_, 0.1 mM ATP) with and without 10 µM CGS9343B or 8 µg/ml calmodulin or control antibody. After centrifugation at 1,000 × g for 10 min at 4 °C, the pellets and supernatants were subjected to immunoblot analysis.

## References

[1] Kleene R, Schachner M (2004). Glycans and neural cell interactions. Nat. Rev. Neurosci..

[2] Bonfanti L, Theodosis DT (2009). Polysialic acid and activity-dependent synapse remodeling. Cell. Adh. Migr..

[3] Mühlenhoff M, Oltmann-Norden I, Weinhold B, Hildebrandt H, Gerardy-Schahn R (2009). Brain development needs sugar: the role of polysialic acid in controlling NCAM functions. Biol. Chem..

[4] El Maarouf A, Rutishauser U (2010). Use of PSA-NCAM in repair of the central nervous system. Adv. Exp. Med. Biol..

[5] Colley KJ, Kitajima K, Sato C (2014). Polysialic acid: biosynthesis, novel functions and applications. Crit. Rev. Biochem. Mol. Biol..

[6] Hildebrandt H, Mühlenhoff M, Gerardy-Schahn R (2010). Polysialylation of NCAM. Adv. Exp. Med. Biol..

[7] Hildebrandt H, Dityatev A (2015). Polysialic acid in brain development and synaptic plasticity. Top. Curr. Chem..

[8] Mishra B (2010). Functional role of the interaction between polysialic acid and extracellular histone H1. J. Neurosci..

[9] Theis T (2013). Functional role of the interaction between polysialic acid and myristoylated alanine-rich C kinase substrate at the plasma membrane. J. Biol. Chem..

[10] Kanato Y, Kitajima K, Sato C (2008). Direct binding of polysialic acid to a brain-derived neurotrophic factor depends on the degree of polymerization. Glycobiology.

[11] Ono S, Hane M, Kitajima K, Sato C (2012). Novel regulation of fibroblast growth factor 2 (FGF2)-mediated cell growth by polysialic acid. J. Biol. Chem..

[12] Endo A (1998). Proteolysis of highly polysialylated NCAM by the tissue plasminogen activator-plasmin system in rats. Neurosc. Lett..

[13] Endo A (1999). Proteolysis of neuronal cell adhesion molecule by the tissue plasminogen activator-plasmin system after kainate injection in the mouse hippocampus. Neurosci. Res..

[14] Hubschmann MV, Skladchikova G, Bock E, Berezin V (2005). Neural cell adhesion molecule function is regulated by metalloproteinase-mediated ectodomain release. J. Neurosci. Res..

[15] Kalus I, Bormann U, Mzoughi M, Schachner M, Kleene R (2006). Proteolytic cleavage of the neural cell adhesion molecule by ADAM17/TACE is involved in neurite outgrowth. J. Neurochem..

[16] Dean RA, Overall CM (2007). Proteomics discovery of metalloproteinase substrates in the cellular context by iTRAQ labeling reveals a diverse MMP-2 substrate degradome. Mol. Cell. Proteomics.

[17] Shichi K (2011). Involvement of matrix metalloproteinase-mediated proteolysis of neural cell adhesion molecule in the development of cerebral ischemic neuronal damage. J. Pharmacol. Exp. Ther..

[18] Kleene R (2010). NCAM-induced neurite outgrowth depends on binding of calmodulin to NCAM and on nuclear import of NCAM and fak fragments. J. Neurosci..

[19] Westphal N, Kleene R, Lutz D, Theis T, Schachner M (2016). Polysialic acid enters the cell nucleus attached to a fragment of the neural cell adhesion molecule NCAM to regulate the circadian rhythm in mouse brain. Mol. Cell. Neurosci..

[20] Huotari J, Helenius A (2011). Endosome maturation. EMBO J..

[21] Lutz D (2012). Generation and nuclear translocation of a sumoylated transmembrane fragment of the cell adhesion molecule L1. J. Biol. Chem..

[22] Verghese GM (1994). Protein kinase C-mediated phosphorylation and calmodulin binding of recombinant myristoylated alanine-rich C kinase substrate (MARCKS) and MARCKS-related protein. J. Biol. Chem..

[23] Gallant C, You JY, Sasaki Y, Grabarek Z, Morgan KG (2005). MARCKS is a major PKC-dependent regulator of calmodulin targeting in smooth muscle. J. Cell Sci..

[24] Niethammer P (2002). Cosignaling of NCAM via lipid rafts and the FGF receptor is required for neuritogenesis. J. Cell. Biol..

[25] Goetz R, Mohammadi M (2013). Exploring mechanisms of FGF signalling through the lens of structural biology. Nat. Rev. Mol. Cell. Biol..

[26] Rauch ME, Ferguson CG, Prestwich GD, Cafiso DS (2002). Myristoylated alanine-rich C kinase substrate (MARCKS) sequesters spin-labeled phosphatidylinositol 4,5-bisphosphate in lipid bilayers. J. Biol. Chem..

[27] Wang J (2002). Lateral sequestration of phosphatidylinositol 4,5-bisphosphate by the basic effector domain of myristoylated alanine-rich C kinase substrate is due to nonspecific electrostatic interactions. J. Biol. Chem..

[28] Ellena JF, Burnitz MC, Cafiso DS (2003). Location of the myristoylated alanine-rich C-kinase substrate (MARCKS) effector domain in negatively charged phospholipid bicelles. Biophys. J..

[29] Gambhir A (2004). Electrostatic sequestration of PIP2 on phospholipid membranes by basic/aromatic regions of proteins. Biophys. J..

[30] Morash SC, Douglas D, McMaster CR, Cook HW, Byers DM (2005). Expression of MARCKS effector domain mutants alters phospholipase D activity and cytoskeletal morphology of SK-N-MC neuroblastoma cells. Neurochem. Res..

[31] Ziemba BP, Burke JE, Masson G, Williams RL, Falke JJ (2016). Regulation of PI3K by PKC and MARCKS: single-molecule analysis of a reconstituted signaling pathway. Biophys. J..

[32] Schmidt HH, Pollock JS, Nakane M, Förstermann U, Murad F (1992). Ca2+/calmodulin-regulated nitric oxide synthases. Cell. Calcium.

[33] Su Z, Blazing MA, Fan D, George SE (1995). The calmodulin-nitric oxide synthase interaction. Critical role of the calmodulin latch domain in enzyme activation. J. Biol. Chem..

[34] Gu Z (2002). S-nitrosylation of matrix metalloproteinases: signaling pathway to neuronal cell death. Science.

[35] Manabe S, Gu Z, Lipton SA (2005). Activation of matrix metalloproteinase-9 via neuronal nitric oxide synthase contributes to NMDA-induced retinal ganglion cell death. Invest. Ophthalmol. Vis. Sci..

[36] Ridnour LA (2007). Nitric oxide regulates matrix metalloproteinase-9 activity by guanylyl-cyclase-dependent and -independent pathways. Proc. Natl. Acad. Sci. USA.

[37] Reich R, Blumenthal M, Liscovitch M (1995). Role of phospholipase D in laminin-induced production of gelatinase A (MMP-2) in metastatic cells. Clin. Exp. Metastasis.

[38] Ispanovic E, Haas TL (2006). JNK and PI3K differentially regulate MMP-2 and MT1-MMP mRNA and protein in response to actin cytoskeleton reorganization in endothelial cells. Am. J. Physiol. Cell. Physiol..

[39] Greif DM, Sacks DB, Michel T (2004). Calmodulin phosphorylation and modulation of endothelial nitric oxide synthase catalysis. Proc. Natl. Acad. Sci. USA.

[40] Secher, T. Soluble NCAM. Structure and function of the neural cell adhesion molecule NCAM (ed. Berezin, V). Advances in Experimental Medicine and Biology, Volume 663, 227–242 (Springer, 2009).

[41] Steinert JR, Chernova T, Forsythe ID (2010). Nitric oxide signaling in brain function, dysfunction, and dementia. Neuroscientist.

[42] Fujioka, H., Dairyo, Y., Yasunaga, K. & Emoto, K. Neural functions of matrix metalloproteinases: plasticity, neurogenesis, and disease. Biochemistry Research International, Volume 2012, Article ID 789083. Hindawi Publishing Corporation (2012). 10.1155/2012/789083 PMC3332068 22567285

[43] Pillai-Nair N (2005). Neural cell adhesion molecule-secreting transgenic mice display abnormalities in GABAergic interneurons and alterations in behavior. J. Neurosci..

[44] Tunçtan B (2002). Circadian variation of nitric oxide synthase activity in mouse tissue. Chronobiol. Int..

[45] Cremer H (1994). Inactivation of the N-CAM gene in mice results in size reduction of the olfactory bulb and deficits in spatial learning. Nature.

[46] Eckhardt M (1995). Molecular characterization of eukaryotic polysialyltransferase-1. Nature.

[47] Mühlenhoff M, Eckhardt M, Bethe A, Frosch M, Gerardy-Schahn R (1996). Polysialylation of NCAM by a single enzyme. Curr. Biol..

[48] Lobaugh LA, Blackshear PJ (1990). Neuropeptide Y stimulation of myosin light chain phosphorylation in cultured aortic smooth muscle cells. J. Biol. Chem..

[49] Frosch M, Görgen I, Boulnois GJ, Timmis KN, Bitter-Suermann D (1985). NZB mouse system for production of monoclonal antibodies to weak bacterial antigens: isolation of an IgG antibody to the polysaccharide capsules of Escherichia coli K1 and group B meningococci. Proc. Natl. Acad. Sci. USA.

[50] Abd El-Hay, S. S. & Colyer, C. L. Development of high-throughput method for measurement of vascular nitric oxide generation in microplate reader. *Molecules* **22**, pii: E127 (2017). 10.3390/molecules22010127 PMC6155585 28098791

[51] St Laurent CD, Moon TC, Befus AD (2015). Measurement of nitric oxide in mast cells with the fluorescent indicator DAF-FM diacetate. Methods Mol. Biol..

[52] Xu Y, Krukoff TL (2007). Adrenomedullin stimulates nitric oxide production from primary rat hypothalamic neurons: roles of calcium and phosphatases. Mol. Pharmacol..

[53] Jaffrey SR, Erdjument-Bromage H, Ferris CD, Tempst P, Snyder SH (2001). Protein S-nitrosylation: a physiological signal for neuronal nitric oxide. Nat. Cell Biol..

